# From trainee to general practitioner: A qualitative study of transition experiences of Flemish GP trainees

**DOI:** 10.1080/13814788.2024.2443603

**Published:** 2025-01-07

**Authors:** Ellen Tisseghem, Joke Fleer, Melissa Horlait, Peter Pype, Emelien Lauwerier

**Affiliations:** a Department of Public Health, Vrije Universiteit Brussel, Brussel, Belgium; b Department of Health Sciences, Section Health Psychology, University Medical Centre Groningen, University of Groningen, The Netherlands; c Department of Public Health and Primary Care, Ghent University, Gent, Belgium; d Interprofessional Collaboration in Education, Research and Practice (IPC-ERP), Department of Public Health and Primary Care, Faculty of Medicine and Health Sciences, Ghent University, Ghent, Belgium; e Department of Experimental-Clinical and Health Psychology, Ghent University; f Department of Psychology, Open University, The Netherlands

**Keywords:** Medical graduate, general practitioners, medical education, professional identity formation

## Abstract

**Background:**

The transition from trainee to professional marks a key milestone in a family doctor’s career, bringing both emotional and mental challenges. This critical period of specialisation shapes how young doctors adapt and influences their future career choices.

**Objectives:**

We explored trainees’ experiences during their first year of advanced medical training in family medicine/general practice, including barriers and facilitators.

**Methods:**

Using qualitative methodology, we conducted ten focus group interviews with 111 trainees. These group interviews were held as part of small-group sessions integrated into a self-guided reflection course. All interviews were held between February 2021 and March 2021, conducted online via MS Teams, recorded, and transcribed verbatim for analysis.

**Findings:**

Our analysis uncovered two adaptational processes during this transition period: personal adaptation and professional socialisation. We interpreted this as a complex balancing act, with impact on mental health aspects such as energy, exhaustion, and self-confidence. Multiple elements at different levels influenced these processes, including the workplace (e.g. interactions with colleagues and patients), the educational program (e.g. assignments, courses), and societal expectations (e.g. role expectations, support).

**Conclusion:**

The findings highlight the importance of understanding both personal adaptation and professional socialisation to support trainees effectively during their transition into practice. Future studies should validate these findings and explore their evolution over time, particularly in relation to adaptation and career choices.

## Key MESSAGES

Adapting during medical training involves managing personal aspirations alongside socialising to professional norms.

Understanding how trainees balance personal and professional growth can optimise support at the workplace, educational program, and societal levels.

Examining adaptation throughout GP careers can provide insights into career challenges and choices.

## Introduction

The World Medical Association General Assembly has recognised physician emotional awareness and well-being as core principles of the professional role, because poor mental health such as distress or burnout can hinder effective patient care [1,2]. Unfortunately, many physicians, including general practitioners (GPs), face an elevated risk of poor mental health compared to the general population and other healthcare professionals [3,4]. GPs, whose expertise lies in generalism, see patients before referral to specialised care, which comes with a high level of responsibility and requires quick, precise decisions in emergencies. Moreover, they serve as the primary point of contact for managing complex care needs, a responsibility set to grow with the increasing number of chronic illnesses [5]. Their broad scope of practice necessitates collaboration with various healthcare providers, navigating interoperable systems, and staying updated with Information and Communication Technology (ICT) advancements, adding to the complexity of their role. Healthy and resilient GPs are required to maintain high-quality care for a society with increasingly complex needs.

This is why medical schools around the world now include mental health care in training [6–8]. The growing attention to well-being, reflected through educational initiatives, is aimed at enhancing professional identity formation (PIF). PIF involves reshaping one’s personal identity through socialisation to align with values and norms associated with the medical profession [9]. A solid PIF is key to producing competent and resilient professionals [10–12]. PIF is a natural evolving and continuous process, influenced by abrupt changes or situational demands, especially during initial working experiences [13,14]. Entering clinical practice prompts re-evaluation of beliefs associated with becoming a doctor, for example; when diagnosing or navigating the daily routine of being a doctor [15,16]. Hence, the socialisation process of transitioning into practice is considered crucial for PIF and GPs resilience in the long term. However there is limited understanding of this period’s intricacies and how PIF evolves. This study explores trainees’ transition to professional GP, focusing on PIF and aspects that aid or hinder it. By exploring trainees’ experiences, we aim to offer insights into fostering resilient GPs, informing preventative efforts in education and early practice.

## Methodology

### Study design

To explore the transition from GP trainee to professional and associated barriers and facilitators, we conducted a qualitative study through focus groups. This method is ideal for exploring new topics in depth [17]. We combined a phenomenological and inductive approach. This allowed us to explore trainees’ experiences on their transition to professional, without being directed by an already established theoretical framework or model.

### Study context

GP training in Flanders, Belgium involves a 3-year advanced medical training in general practice, open to those who have completed a Bachelor and Master in Medicine. Participating GP trainees were in their first year of advanced training combining clinical internship with courses. One course, a guided self-reflection module, focuses on developing self-awareness and critical thinking in practice. This course is held in small groups of 10–15, led by an experienced GP who acts as a tutor, and encourages trainees to reflect on their experiences, clinical decision-making, interactions and professional growth. The focus groups, conducted as part of these small-group sessions, took place from late February to mid-March 2021 and were held online via Microsoft Teams due to pandemic regulations.

### Participant selection

This study was part of a larger mixed-methods project, which included a 1-year longitudinal survey, focus group interviews, and parallel longitudinal interviews among a large sample of trainees in their first year of advanced training in general practice. Here, we present findings solely from the focus groups. Details on characteristics of the larger sample are available and will be published elsewhere. Before the start of the small-group sessions, tutors who agreed to participate in the study approached their trainees and obtained written informed consent for this study. Ten focus groups were conducted, totalling 111 GP trainees (see Table 1).

**Table 1.** Composition of the focus groups.

Focus group	GP tutor	Number of participants	Male participants	Female Participants	Duration (min)
1	A.	12	2	10	50
2	B.	10	2	8	45
3	C.	7	2	5	61
4	C.	7	3	4	78
5	D.	12	5	7	43
6	E.	12	1	11	51
7	F.	13	3	10	78
8	G.	11	4	7	48
9	H.	13*	4*	9*	52
10	I.	14*	4*	10*	70
Total		111	30	81	576

* These figures reflect the formal composition of the groups. The actual composition (total number of participants, proportion of male and female participants could not be ascertained since only the audio file and no visual material was recorded by the tutor).

### Data collection

A semi-structured guide was collaboratively developed by the research team, to facilitate focus group discussions on mental health during the transition from GP trainee to professional. No specific theoretical framework was employed, but the guide was informed by key concepts, namely mental health, adaptation, transition into practice, and barriers and facilitators. These concepts were translated into sample questions by the research team. Tutors were provided with this list, to help them guide the discussions (see Appendix: Interview Guide). Focus groups averaged 58 min each, with video recordings transcribed verbatim for analysis.

### Data analysis

Data analysis, following the Constructivist Grounded Theory (CGT) approach – an inductive method that generates theories from data rather than pre-existing frameworks – involved several phases and close interaction among the multidisciplinary team, which gathered different disciplines (GP, psychology) and complementary expertise (clinical-, research- and policy-related). The team included a GP and medical sciences expert (PP), (health) psychologists (ET, MH, JF, EL), and a health policy expert (MH). After transcription, a first focus group interview was read in detail by all members to acquire an overall picture. Particular detail was given to mental health and transitioning to professional life. During a first meeting, insights of members were shared and an initial coding tree was created. A second focus group transcript was read alike, and during a second meeting, team members discussed initial codes and added others. The remaining transcripts were coded by two members of the project team separately (ET, EL), and subsequently discussed among the team. This led to the identification of emerging themes, possible meanings of experiences, and their interconnectedness. ET and EL further refined these themes by reviewing the transcripts. After verification and refinement, a visual scheme was drafted and discussed with all team members, including JF, who was not previously involved, until consensus was reached.

## Results

Two parallel adaptational processes emerged from the data that related to transition into GP practice: one relating to life goals and personal aspirations and the other relating to becoming a professional. Furthermore, trainees experienced influences at the level of the workplace, the curriculum and society that hindered or facilitated adaptation. Below, we describe these per level.

### Two processes at the heart of transitioning into practice: A complex balancing act

The focus group analysis of GP trainees’ transition experiences revealed two key adaptational processes: personal adaptation, i.e. striving towards personal goals and aspirations, and professional socialisation, i.e. becoming a professional by internalising work-related norms and expectations as one’s own. Both processes were central to this critical first year of advanced medical training where trainees transition to becoming professional GPs, as evident from all focus groups. For most trainees, balancing these two clearly was a work in progress, but approached with growing confidence. Some trainees noted that the transition period was crucial for building confidence and personal growth, leading to a sense of tranquillity and coherence in their lives.

I think you are a lot more mature, (…). Indeed, you let go of that part of being a student, and now the adult life has started. (…). I think that this (maturity) is also partly due to the training, a kind of personal growth that you also notice outside the education. (Focus group 2)

I do think you are a more confident in everything you do(…) I didn’t expect to learn so many things so quickly and I really like that, yes. You do feel more confident at work and that translates into your private life. That you are less stressed and so on. (Focus group 6)

However, emotional and psychological disturbances were often mentioned. Some trainees expressed concerns about excessive clinical responsibility (too) early in their careers, including fear of mistakes. The added burden of responsibilities outside of work often exacerbated these feelings, making this phase particularly overwhelming for some.

You suddenly have responsibility over a lot of things. At work, earning your own living. Most people also spent years in student homes, they are no longer able to stay at mother’s hotel. It gives you a bit more independence and then there is the added burden of a house and a household and a car and all those responsibilities at once. It was quite an adjustment for me. (Focus group 8)

Some trainees reported positive evolution over time by trying out strategies to lower demands concerning personal goals to save energy for work, or vice versa.

I do feel that I am more self-confident in practice, which is a positive development that I have noticed, and I have the impression that, whereas I used to be a very punctual person, I can put that aside a bit more now because there is a high work pressure and you just don’t always get everything completed one hundred percent before you go home and then you have to work on it again at home which drains your energy. And I have learned to let go of that, so I don’t think it has been such a negative evolution. (Focus group 3)

Others struggled to balance both processes, leading to increased tension and a less joyful transition. Work encroaching on personal time left little room for relaxation, negatively affecting mental health. For some, maintaining balance was challenging, with many describing the recent period as energy-depleting due to adaptation difficulties.

When I get home in the evening I don’t have any energy left to do it, because you’ve had to concentrate all day and then you should do it in the evening or at the weekend… But on the weekends I notice I want to recharge. So sometimes I find it a bit too much with all those tasks and preparations. You also regularly have to prepare a case in practice, and I actually never have any time for that during the week. I can’t demarcate it during my hours, thus I find it very difficult to make time for it. I find it very difficult to maintain the balance between your work and your home life. (Focus group 5)

### Elements that hinder or facilitate adaptation at different levels

**Figure 1.**
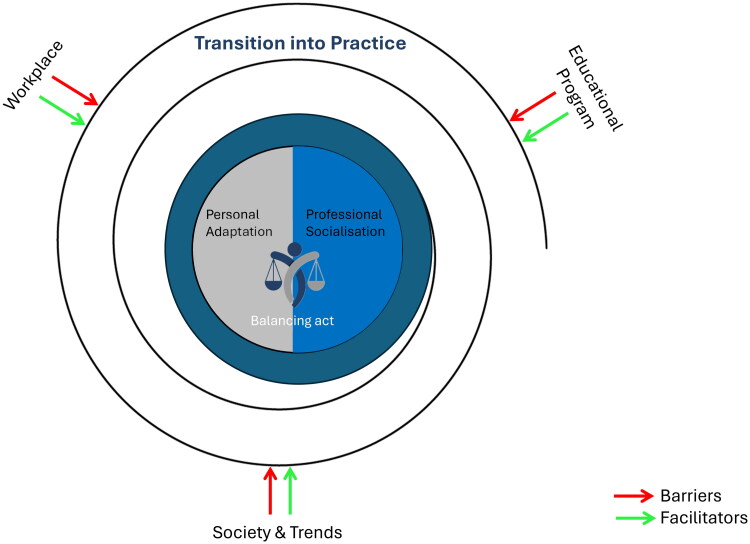
Visual representation of the findings showing a balancing act of two adaptational processes, namely personal adaptation and professional socialisation, at the heart of the transition into practice as a trainee. The arrows represent elements that influence this transition on different levels (educational program, workplace, society & trends). At each level, elements can both hinder (barriers: in red) and facilitate (facilitators: in green) the adaptational processes.

The success of both adaptational processes – professional socialisation and personal adaptation – was affected by various elements across the workplace, educational program, and society (see Figure 1).

#### The workplace level

The interplay was most evident in the workplace, as trainees strived to become part of the team while simultaneously pursuing their own self-discovery. This involved both formal aspects, like becoming skilled doctors through experience, and informal aspects, such as engaging in workplace interactions.

##### Organising and managing practice

This transitional phase highlighted GP trainees’ desire to integrate into the team, manage their practice, and make confident decisions. However, they quickly felt overwhelmed by new challenges, such as unpredictable interruptions and emergencies, which disrupted their schedules and led them to question their future task management.

Well, as it is now it’s not so bad, but later I would like to work by appointment more, because then you have a bit more of an idea of where you will end up. Well, now sometimes it’s (consultations) quickly done and sometimes it takes longer, and that’s a bit unpredictable. (Focus group 3)

Some trainees found the lack of control almost unbearable, others saw it as a natural part of learning to manage the practice. As some noticed, the challenges associated with the transition to professional were often more prominent at the beginning of the internship, but diminished over time.

I have noticed that I am more self-assured now than in the first few months and that in itself is a nice feeling. Also at work, because you don’t have to call your colleagues so much to ask questions, and afterwards you really feel good that you did the consultation correctly. (Focus group 2)

##### Building connections

According to the GP trainees, the culture of the practice and interactions with colleagues, supervisors, and patients significantly impacted their mental health, either facilitating or hindering personal and professional growth. A positive atmosphere allowed trainees to show their vulnerability, be authentic and to ventilate about challenging cases. Which in turn created a solid ground for personal and professional growth.

For me, the atmosphere between everyone is actually very good. (…) There is always a lot of laughter. The whole atmosphere, and the openness between everyone, and the fact that I was one of the team right from the start. That gives me a lot of energy. (Focus group 2)

The interaction with patients, and then certainly when they compliment you, but also when your PS (practice supervisor) confirms you or compliments you on how you did, that’s always energising, I think, because then you really feel that you’re doing a good job. (Focus group 4)

However interactions were sometimes seen as energy draining, with trainees feeling a gap between external expectations and their personal and professional capabilities.

So I don’t mind giving a lot of energy for patients or helping them, but if you’re giving a lot, or trying to help out and that patient puts up a wall, and gives very little back, also in communication itself, saying very little. When you have to force everything out like that, I find that a consultation lasts very long and that I have to recover my energy during the following consultations. (Focus group 1)

#### The educational program

Many trainees felt disconnected from their ‘trainee status’ despite ongoing educational tasks. They were eager to transition to professional GP and resisted being seen only as trainees. A desire partly driven by personal aspirations, as trainees associated the transition phase with entering ‘adult life’, where new and greater responsibilities emerge. It also related to professional socialisation, with trainees experiencing conflicts between the educational program’s expectations (e.g. courses, assignments, thesis) and the desire to focus on the challenges arising from their recent practical experiences.

We still have lessons and tasks, but I don’t feel like a real student anymore, I feel more like a full-fledged doctor who still has to learn a lot, but at the moment I no longer see myself as a student. (Focus group 3)

I do think he is right about those silly assignments and I do think the course should be evaluated on how you are progressing, but I think it could be done in a less stressful way for the students, (…), all those additional tasks, I don’t know if they are useful per se and evaluate how you are doing. You have to put in a lot of time besides your work. I would have preferred us to be ‘ordinary working people’… (Focus Group 7)

#### The societal level

Societal trends influenced the transition, including changes in GP`s roles, tasks and requirements, and mental health support networks.

##### Evolution of general practitioner roles, tasks, and requirements

Besides more general societal trends like the constant flow of information and digital connectedness, some trainees mentioned changes specific to the GP profession. One concerned changing views of the GP as more progressive and aligned with modern needs. For example, GP trainees mentioned being trained to prioritise work–life balance differently than past generations, yet they perceived limited support or recognition to facilitate this in practice.

It’s always something that they’ve emphasised; we need to find a balance. But actually very little is done to make that easier for us. I noticed that during my training, ‘Make sure that in your internship year you sufficiently…’ and so on, but when it came down to it, and you asked for a vacation, it was always very difficult. (…) I am a member of a GP in training advisory group, and if you raise the issue of ‘this could be better in our program’, you’re often told… ‘Yes, it all used to be worse’… Then I think, no, we shouldn’t go there. Now you’re all saying that we need to take better care of ourselves and you’re actually still ensuring that there are few opportunities to be able to do that. (Focus group 1)

Trainees identified several requirements, roles, and tasks as burdens that hindered their ability to perform their GP role with excitement. These included high-burden administrative tasks, the difficult match between educational demands and clinical practice, rising patient expectations, the increasing complexity of cases due to the growing prevalence of chronic diseases, and challenges in task division and role alignment as a result of multidisciplinary collaboration. The pandemic exacerbated these issues, particularly the rise in repetitive administrative tasks and high work pressure, leading to greater work–life imbalance. This burden led some trainees to question if this still aligned with their personal desires and other roles they wanted to take up beside work, and if the costs weigh the benefits.

But also because of corona (…) I have the feeling that the role I took on, in which I felt best as a person, the role of friend and organizer, etc. I really enjoyed doing that, and now that has been completely lost and replaced by the role of doctor, in which a lot is asked of you, both by my supervisor as well as by the patient. I have to say that sometimes I do have a really hard time with the fact that you can’t take on those other roles in this time. (Focus group 1)

##### Mental health support networks

Generally, trainees expressed satisfaction with the level of support from their personal network and/or social context. Many found the support from colleagues, supervisors, family, partners, or peers crucial in maintaining (mental) well-being during the transition to GP trainee. However, despite relying on personal networks for mental health issues, they faced barriers seeking professional help, often because they felt like they were failing as GPs when doing so.

I do know for example that I can go to you (Tutor) for problems with the training and that I am so lucky that I still live at home and have a very warm family, where I can always turn to. (Focus group 1)

I honestly think I would have a hard time with it [seeking professional mental health support]. Just because I don’t quite know where and how and (…) I think that wouldn’t be easy for me, because I would feel like, I was failing a bit as a good doctor because doctors should know how to deal with it, so to speak. So I think I would have a hard time dealing with that. (Focus group 5)

## Discussion

### Main findings

This study aimed to understand how GP trainees transition to professional GP by identifying elements that facilitate or hinder this process. From our results we identified two adaptational processes at the heart of the first-year transition to professional GP: striving towards personal goals and aspirations, and professional socialisation, where trainees align work goals with personal values. We interpreted these processes as a balancing act, with successful completion contributing to improved mental health outcomes. While we did not measure mental health directly, trainees discussed these processes in relation to different mental health aspects such as energy, exhaustion, or self-confidence.

The identified process of professional socialisation aligns with literature on PIF, which highlights that early clinical experiences intensify work identity development. PIF is a developmental framework specific to the context of (medical) professional work where trainees gradually merge personal values with professional ones [9,18]. Successful PIF allows medical trainees to fully embody their roles as medics [19]. Our qualitative study supports previous findings that this transition phase, while stressful, is a natural progression tied to first clinical encounters [20,21]. Trainees must adapt to these new practices, for which they perceive themselves not yet capable or knowledgeable, leading to temporary work–life imbalances [22]. Resulting emotional disturbances seemed natural, inevitable and inherent to the transition, and resided from the process of active exploration. According to the identity status theory of Marcia [23], active exploration is a phase in which an individual explores new commitments and roles in order to build a coherent sense of self, integrating professional and personal aspects. However, active exploration is just the first step towards this integration, typically unfolding over an extended period as conflicting identities or roles are reconciled [23]. Future research should explore when and how this leads to positive adaptation.

Furthermore, our findings underscore the importance of achieving personal standards and goals in addition to professional socialisation during the transition to professional. Personal development extends beyond adolescence and is critical in this phase. Unlike traditional frameworks of PIF, which assume alignment with a pre-existing self, our results suggest that trainees actively reshape their identities to fit evolving professional roles [9]. Exposure to work tasks appear to bring personal aspirations to the front, requiring individuals to invest time and energy in pursuing or reassessing these goals. It might be this dynamic balancing act, rather than socialisation alone, that is crucial for successful transitioning. Future research should examine how this reshaping process evolves over a career, particularly in relation to conflicting goals and aspirations. Integrating psychological theories, like Carver and Scheier’s theory Self-regulation Model and Brandstädter & Renner’s Life-Span Theory, with professional socialisation theories can offer a fuller understanding of these complex transition dynamics [24,25].

Additionally, we found that elements at the workplace, educational program, and societal levels can either support or hinder adaptation, as is also emphasised by Cruess’ PIF Theory [9]. Supportive mentorship and inclusive cultures facilitate identity integration, while hierarchical structures and stigma can impede it [20]. Our findings underline the importance of multi-level influences on PIF and suggest that ecological approaches in future research could be valuable. The Ecological Systems Theory highlights that interconnected systems, from immediate (micro) to broader (meso and macro) contexts, influence individuals [26]. For example, a (mis-)alignment between the educational program and clinical practice may impact balance and affect trainees’ adaptability. Also, discrepancies may exist between healthcare policies trying to facilitate a work–life balance and the reality of daily clinical practice.

### Strengths and limitations

We used qualitative methods to explore trainees’ early practice experiences in depth. The focus groups’ strength lies in their intimate setting and the trust established with the tutor, enhancing the credibility of the findings. However, varying interpretations by different interviewers may introduce biases, affecting data collection and analysis. To minimise these risks, we provided clear guidelines and standardised interview instructions for the tutors [27]. Another limitation is the risk of peer conformity as participants knew each other. While this risk cannot be fully eliminated, the range of responses suggests the experienced tutors facilitated diverse perspectives.

A second strength is our large sample of first-year Flemish GP trainees, from an interuniversity program across all four Dutch-speaking medical schools, providing a comprehensive view of their experiences during a critical transition period, enhancing the transferability of our findings. However, the results may not be fully generalisable to trainees in later years or those from other medical disciplines, highlighting the need for further studies with more diverse samples. Third, the interviews were conducted during the COVID-19 pandemic, which may have amplified certain experiences, bringing to light issues that might otherwise remain hidden. Some of these experiences may persist across contexts, but future studies should confirm this. Longitudinal cohort studies could provide a clearer picture of how these experiences evolve.

### Implications

Our results offer insights into trainees’ transition to becoming a professional and suggest practical implications. Personal adaptation is as important as socialisation during this transition. Trainees must adjust to their new professional role as well as adult responsibilities; balancing mental well-being aspects like energy, exhaustion, and self-confidence. Future research should examine why some trainees struggle, key turning points, and their impact on career adaptation. Early interventions should include self-care, peer support, tailored training, and systemic improvements. Workplace instructors must recognise the steep learning curve and the challenges of balancing work and study. Educational institutions and clinical practices should ensure time off and prevent overload through flexible training programs that match the reality of clinical practice. Transitioning to a doctor involves a multi-layered system of personal, practice, curricular, and societal factors, all of which must be addressed simultaneously. Additionally, cultural norms, like stigma around seeking help and pressure to avoid mistakes, can hinder well-being. Understanding how these norms are shaped can foster a more supportive environment.

## Conclusion

The transition from trainee to professional requires navigating a delicate balance between pursuing personal aspirations and adherence to professional norms, all within the context of the workplace, educational program and broader societal expectations. Understanding and addressing this interplay is crucial for supporting the mental well-being and professional socialisation of GPs in training, and may provide further directions for tailored interventions.

## Supplementary Material

Supplemental Material

## Data Availability

The participants of this study did not give written consent for their data to be shared publicly, and due to the sensitive nature of the research and ethical restrictions, supporting data is not available.

## Appendix. Interview guide

Sample questions used as a basis for the focus group interviews. Tutors were given specific instructions through e-mail accompanying these topics. It was accentuated to have an open, non-formal discussion in which the questions below could help to bring in some direction in case trainees do not come up with some of this input themselves. The tutors were asked to guide the interview, but to not interfere with their own opinions or advice in order for the discussion to be as openly as possible. We also indicated that any other topics related to their mental health, adaptation and transition into practice could also be discussed or elaborated, even if not displayed in this list.

- Which factors give you a boost of energy in the workplace?
- Which ones bring your energy level down?
- What gives you satisfaction?
- What stress factors do you experience during the training?
- How do you deal with this?
- Which things influence your self-confidence as a doctor in a positive/negative way?
- What is your relationship with your colleagues and your practical trainer?
- What do you think of the work-life-study balance?
- Do you feel that you are sufficiently supported when things do not go well?
- Where can you go for support if things don’t go well?
