# Supplementary material for: From trainee to general practitioner: A qualitative study of transition experiences of Flemish GP trainees
Source: Eur J Gen Pract. 2025 Jan 7;31(1):2443603. doi: 10.1080/13814788.2024.2443603 (PMC11722024; doi:10.1080/13814788.2024.2443603)
Supplement: Supplemental Material [file IGEN_A_2443603_SM1821.docx]

## ***Interview Guide***

Sample questions used as a basis for the focus group interviews. Tutors were given specific instructions through e-mail accompanying these topics. It was accentuated to have an open, non-formal discussion in which the questions below could help to bring in some direction in case trainees do not come up with some of this input themselves. The tutors were asked to guide the interview, but to not interfere with their own opinions or advice in order for the discussion to be as openly as possible. We also indicated that any other topics related to their mental health, adaptation and transition into practice could also be discussed or elaborated, even if not displayed in this list.

- Which factors give you a boost of energy in the workplace?
- Which ones bring your energy level down?
- What gives you satisfaction?
- What stress factors do you experience during the training?
- How do you deal with this?
- Which things influence your self-confidence as a doctor in a positive/negative way?
- What is your relationship with your colleagues and your practical trainer?
- What do you think of the work-life-study balance?
- Do you feel that you are sufficiently supported when things do not go well?
- Where can you go for support if things don't go well?
